# Supplementary material for: Adapting antibacterial display to identify serum-active macrocyclic peptide antibiotics
Source: PNAS Nexus. 2023 Aug 17;2(8):pgad270. doi: 10.1093/pnasnexus/pgad270 (PMC10449418; doi:10.1093/pnasnexus/pgad270)
Supplement: pgad270_Supplementary_Data [file pgad270_supplementary_data.zip › PNASNEXUS-PNASNEXUS-2023-00665-T-s03.pdf]

Supplemental material for

Adapting antibacterial display to identify serum active macrocyclic peptide antibiotics

Justin R. Randall, Kyra E. Groover, Angela C. O'Donnell, Joseph M. Garza, T. Jeffrey Cole, and Bryan W. Davies

Correspondence to:

Justin Randall (justrand@utexas.edu) or Bryan Davies (bwdavies@utexas.edu)

This PDF includes:

Supplemental Figures S1-4
Supplemental Tables S1-S6

A

KNOWN B-L-B AMPS		#AA	Charged AAs
POLYPHEMUSIN-1	RRWCFRVCYRGFCYRKCR	18	7
TIGERININ-1	FCTMIPIPRCY	11	1
BECTENECIN	RLCRIVVIRVCR	12	4
THANATIN	GSKKPVPIIYCNRRTGKCQRM	21	6
ARENICIN-1	RWCVYAYVRIRGVLVRYRRCW	21	6
TACHYPLESIN-1	KWCFRVCYRGICYRR	17	6
GOMESIN	CRRLCYKQRCVTYCRGR	18	6
ANDROCTONIN	RSVCRQIKICRRRGGCYYKCTNRPY	25	8
PROTEGRIN-1	RGGRLCYCRRRFCVCVGR	18	6
DEFENSIN-1	GFCRCLCRRGVCRCICTR	17	5
	Averages	17.9	5.6
	Library	14,16,18	2-13

B

AA	Frequency		AA	Frequency		AA	Frequency	
Tails	Natural	Library	Sheets	Natural	Library	Loop	Natural	Library
R	0.31	0.40	C	0.36	0.30	R	0.32	0.52
G	0.10	0.17	R	0.20	0.16	G	0.15	0.19
K	0.07	0.10	Y	0.09	0.10	I	0.11	0.15
V	0.07	-	V	0.09	0.10	V	0.09	0.06
F	0.07	0.06	K	0.05	0.07	K	0.06	0.08
Y	0.07	0.04	I	0.04	0.05	Y	0.06	-
W	0.07	0.04	L	0.03	0.04	F	0.04	-
P	0.05	-	T	0.03	0.03	L	0.04	-
T	0.03	0.04	F	0.02	0.04	M	0.04	-
I	0.03	-	W	0.02	0.02	P	0.04	-
S	0.03	-	A	0.02	-	Q	0.02	-
L	0.03	-	Q	0.01	-	T	0.02	-
N	0.02	-	P	0.01	-			
A	0.02	-	S	0.01	-			
M	0.02	-	G	0.01	-			

C

Position	0	1	2	3	4	5	6	7	8	9	10	11	12	13	14	15	16	17
AA(s)	R	CGR	RK	C	V	CY	RIGV	R	GR	RKI	CR	RL	C	YF	CYW[S]	RG	RG[S]	RTK
# Possible	1	3	2	1	1	2	4	1	2	3	2	2	1	2	3	2	2	3
Charge	1	0.33	1				0.25	1	0.5	0.66	0.5	0.5				0.5	0.5	0.66
Codon	**AGG**	**BGC**	**ARA**	**TGC**	**GTT**	**TRT**	**RKA**	**AGG**	**RGA**	**ADA**	**YGC**	**CKA**	**TGC**	**TWC**	**TRK**	**RGA**	**BGA**	**AVA**
		CGC	AAA			TGT	AGA		AGA	AGA	TGC	CGA	TGC	TAC	TAG	GGA	CGA	AGA
		TGC	AGA			TAT	ATA		GGA	AAA	CGC	CTA		TTC	TAT	AGA	TGA	AAA
		GGC					GGA			ATA					TGT		GGA	ACA
							GTA								TGG			

Reverse primer TBT TCV TCY MYA GWA GCA TMG GCR THT TCY CCT TMY AYA AAC GCA TYT GCV CCT

	14aa	16aa	18aa		
Length	14aa	16aa	18aa		
Average charge	5.74	6.24	7.4		
Unique peptides	2304	13824	82944	Total	99,072

Figure S1: Design of a peptide library based on natural beta-AMP residue frequency. A) Amino acid sequences for ten natural beta-AMP sequences. B) individual residue frequencies found in the tail, sheet, and loop regions of the ten-natural beta-AMPs and the BH peptide library. C) Codons used to cod for amino acids at each position of the BH peptide library.

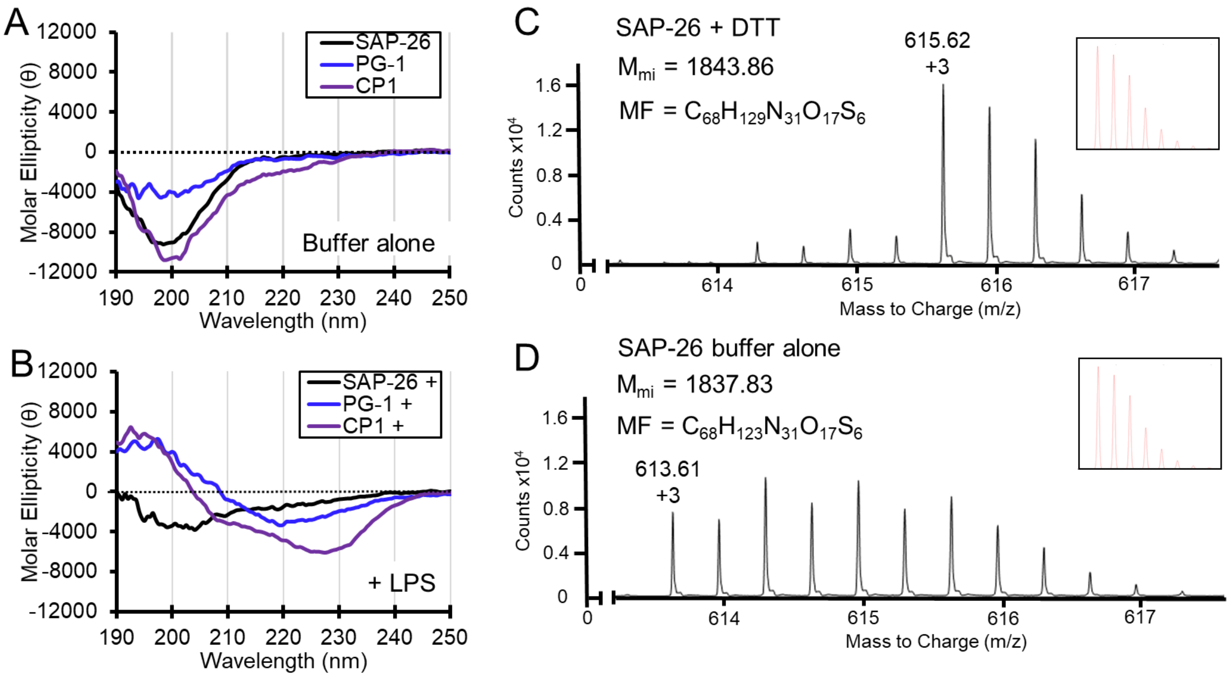

Figure S2: SAP-26 is an unstructured peptide macrocycle. Circular dichroism spectra of SAP-26, Protegrin-1 (PG-1), and Cecropin P1 (CP1) in buffer (A) with 0.2 mg/ml LPS (B). Data represents the mean of three technical replicates. High-resolution mass spectrometry of SAP-26 showing individual isotopes with 10 mM DTT (C) or in buffer alone (D). Mass to charge and charge state are highlighted for the peak corresponding to the monoisotopic mass (M_{mi}). The calculated M_{mi} and molecular formula (MF) are shown and expected isotope distribution is inset.

A

***E. coli* 25922 antibiotic killing in PBS glucose**

Antibiotic	MBC (µg/ml)
SAP-26	1
Protegrin-1	2
Cecropin P1	2
Kanamycin	1

MBC: minimum bactericidal concentration,

B

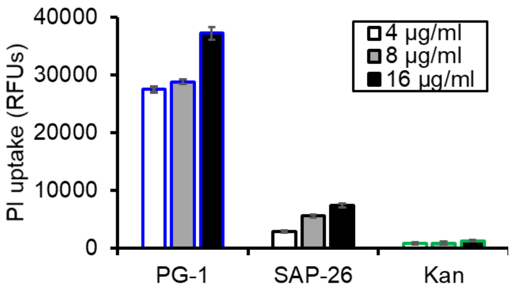

Figure S3: Comparing SAP-26 activity to other antibiotics. A) Table showing the MBC of antibiotics used in Fig. 3 in PBS supplemented with 50 mM glucose. B) PI fluorescence at various concentrations for cells treated with Protegrin-1 (PG-1), SAP-26, and Kanamycin (Kan) after two hours.

A

Effect of colistin resistance on SAP-26

Strain	SAP-26 MIC (µg/ml)	Colistin MIC (µg/ml)
E. coli W3110	1	0.5
+ pDM1_empty	1	0.5
+ pDM1_mcr-1	1	8

MH: Mueller-Hinton, MIC: minimum bactericidal concentration

B

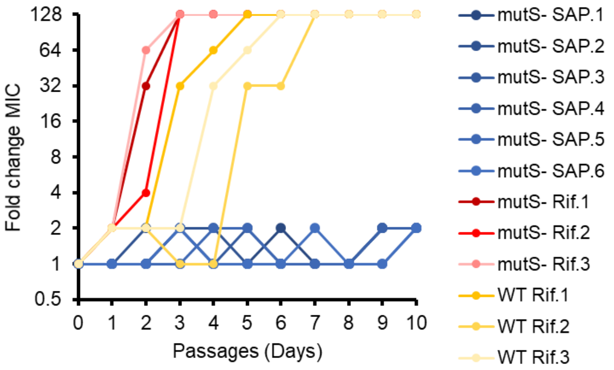

C

Beta-lactam sensitive knockout MICs.

Antibiotic	Keio WT MIC (ug/ml)	*dacA::kan* MIC (ug/ml)	*cpoB::kan* MIC (ug/ml)	*mrcB::kan* MIC (ug/ml)
SAP-26	1	1	2	1
Protegrin-1	8	8	8	8
Carb	8	1	≤ 1	2
Cefuroxime	8	2	1	1

MIC: minimum inhibitory concentration, Carb: carbenicillin

Figure S4: SAP-26 resistance and cell wall deficient insensitivity. A) Table showing SAP-26 activity against E. coli W3110 containing an IPTG inducible empty vector or mcr-1 expressing plasmids. IPTG was added to 0.5 mM. B) Graph showing the Fold-change in MIC for independent isolates of *E. coli* K12 (WT) and *mutS* deficient (mutS-) strains during ten days of serial passage in sub inhibitory concentrations of SAP-26 (SAP) or Rifampin (Rif). C) Table showing the MIC of antibiotics against strains deficient in cell wall synthesis.

Table S1. Effect of bacterial strain and media conditions on CAMP activity.

			E. coli W3110 MBC (μg/ml)	*E. coli* 25922 MBC (μg/ml)	
**CAMP**	**Source**	**Structure**	**Mueller-Hinton**	**Mueller-Hinton**	**Human Serum**
PepC	SLAY	α-helical	32	64	>256
Symbah-1	SLAY	β-hairpin	16	32	>256
SySA-5	SLAY	β-hairpin	16	64	>256
Melittin	Nature	α-helical	16	8	>256
Cecropin P1	Nature	α-helical	2	2	32
Protegrin-1	Nature	β-hairpin	4	8	32
Tachyplesin-1	Nature	β-hairpin	4	8	16

CAMP: cationic antimicrobial peptide, MBC: minimum bactericidal concentration

Table S2. Characteristics of randomly selected BH library peptides.

Name	Sequence	MBC MH	MBC HS
BHR-1	RCKCVYRRRIRRCFCRRR	8	>128
BHR-2	RGKCVYGRRRRRCYWRGR	64	>128
BHR-3	RRKCVCGRRICRCYCG	>128	>128
BHR-4	RRKCVYIRRIRRCYWRGT	8	128
BHR-5	RRRCVYRRRKCRCYWGGT	64	>128
BHR-6	RGKCVCIRGRRLCYYR	32	>128
BHR-7	RRRCVYIRGICRCFYGGR	32	>128
BHR-8	RRKCVCGRRKRLCYYRRK	64	>128
BHR-9	RGRCVCRRGICLCYWRRK	4	>128
BHR-10	RCRCVCVRGKRRCFWGGK	128	>128
BHR-11	RGRCVYRRGICLCFYGRR	32	>128
BHR-12	RGKCVYIRGICLCYYGGT	>128	>128
BHR-13	RRKCVYRRRKCLCYWR	64	>128
BHR-14	RCRCVCGRGRRRCYCRRK	64	>128
BHR-15	RCKCVYVRGKCRCFCGGR	64	>128
BHR-16	RRKCVCIRRICLCYCGGR	128	>128
BHR-17	RCKCVYVRRRCRCYYG	64	>128
BHR-18	RRRCVCVRRRCLCFYRRR	128	>128
BHR-19	RGKCVCVRRIRLCFCG	4	>128
BHR-20	RGKCVCRRGKRRCYYGRR	128	>128
BHR-21	RGRCVCIRGRRLCYWGGK	>128	>128
BHR-22	RRKCVYIRGKRRCYWGRK	128	>128
BHR-23	RCKCVCIRGRCRCFWRGK	32	>128
BHR-24	RGKCVCIRRKRRCYCRRK	128	>128
**% Active**	–	**87.5**	**4.0**
**Median MBC**	–	**64**	**128**

MBC: minimum bactericidal concentration, MH: Mueller-Hinton;
HS: Human Serum

Table S3: SLAY active BH peptide biochemical characteristics.

Name	Sequence	MH MBC	HS MBC	L2FC	Length	#S-S	Charge
BHS-1	RRRCVYRRRRRRCFCRRR	64	>128	-1.05	18	1	11.88
BHS-2	RCKCVYGRGICLCFCGGR	128	>128	-1.15	18	nd	3.82
BHS-3	RRRCVCVRRRRRCFCRRR	64	>128	-1.15	18	2	10.85
BHS-4	RGRCVYVRRIRLCYYG	16	>128	-0.57	16	1	4.91
BHS-5	RRRCVYVRGICLCYCRRR	16	128	-0.87	18	nd	6.85
BHS-6	RGRCVYVRGKRLCYWRRR	8	>128	-0.97	18	1	7.91
BHS-7	RGKCVCIRRICRCYWGRT	2	>128	-0.83	18	2	5.85
BHS-8	RGKCVCRRGRRLCFCGGK	>128	>128	-1.03	18	2	6.85
BHS-9	RGRCVCVRGKCRCYWRRK	8	128	-1.05	18	2	7.85
BHS-10	RGRCVCRRRRCRCY	128	>128	-1.15	14	2	6.85
BHS-11	RCRCVYGRGRRLCFWR	4	128	-1.31	16	1	5.88
BHS-12	RGKCVYRRGKCRCFWR	32	128	-1.33	16	nd	6.88
BHS-13	RCRCVCRRRKCRCFWR	8	>128	-1.37	16	2	7.82
BHS-14	RRRCVCIRRKRLCFCG	2	64	-1.21	16	2	6.85
BHS-15	RCRCVYVRRKRRCYYRGR	64	>128	-1.29	18	1	8.88
BHS-16	RCKCVYIRRRRLCYCRGR	2	64	-1.44	18	2	7.85
BHS-17	RCRCVCIRRRRRCFCGGK	32	>128	-1.57	18	2	7.82
BHS-18	RCRCVCRRRKCRCYCGRT	1	32	-1.28	18	3	7.79
BHS-19	RCRCVYIRRRRRCF	64	>128	-1.16	14	1	6.88
BHS-20	RGKCVCRRRRCRCYWR	4	128	-1.52	16	2	7.85
BHS-21	RGRCVCIRRRCRCYYRGR	8	128	-1.58	18	2	7.85
BHS-22	RGRCVCRRRKCRCYCGGR	64	>128	-1.7	18	nd	7.82
BHS-23	RCKCVCRRRRCRCYWRGR	16	>128	-1.58	18	nd	8.82
BHS-24	RCRCVCRRGKRRCFCRRR	128	>128	-1.63	18	2	9.82
BHS-25	RCKCVCIRRKCRCFCRGK	8	>128	-1.34	18	3	7.79
BHS-26	RCKCVCIRRKCRCYCRGK	8	128	-1.4	18	3	7.79
BHS-27	RGRCVCRRRRRRCF	128	>128	-0.95	14	nd	7.88
BHS-28	RCRCVYRRRRRRCFCR	32	>128	-0.99	16	2	8.85
BHS-29	RCRCVYRRRRRRCYCR	16	128	-0.61	16	2	8.85
BHS-30	RGRCVCGRRRCLCYYRRR	16	>128	-0.67	18	2	7.85
BHS-31	RCRCVCRRRRCRCFWR	16	>128	-1.43	16	2	7.82
BHS-32	RCKCVYRRRRRRCFCR	64	>128	-1.23	16	2	8.85
BHS-33	RRKCVYIRRRRRCYWGGR	32	>128	-1.35	18	1	8.91
BHS-34	RRRCVYRRGRRLCYWGGR	64	>128	-1.09	18	1	7.91
BHS-35	RCRCVCGRRKRRCYCGGK	128	>128	-1.33	18	2	7.82
BHS-36	RGRCVCRRRKCRCYWGRT	4	64	-1.2	18	2	7.85
BHS-37	RCKCVYIRRRRRCFCRGR	32	>128	-1.59	18	2	8.85
BHS-38	RGRCVCGRRRCRCYYRRR	8	>128	-0.96	18	2	8.85
BHS-39	RGRCVCRRRKCRCFWRGK	32	128	-1.29	18	2	8.85
BHS-40	RCKCVCRRRRRRCFCGGK	128	>128	-1.81	18	2	8.82
BHS-41	RCRCVCRRRRCRCYCRGT	4	64	-1.35	18	3	7.79

MBC: minimum bactercidal information, MH: Meuller-Hinton, HS: human serum, L2FC: log_2-fold change in reads, #S-S: number of disulfide bonds found in the majority of molecular population

Table S4: SAP serum optimization library.

Name	Sequence	MH MBC (µg/ml)	HS MBC (µg/ml)	%Hemo
SAP	RCRCVCRRRKCRCYCGRT	4	64	nd
SAP-1	RCRCVCRRRKCLCQCRRT	4	64	0.1 ± 0.2
SAP-2	RCRCVCRRRKCLCQCRRT^{A}	4	32	0.2 ± 0.1
SAP-3	RCRCVCRRRKCLCQCRR-	16	128	-0.1 ± 0.0
SAP-4	-CRCVCRRRKCLCQCRRT	4	128	6.4 ± 0.5
SAP-5	-CRCVCRRRKCLCQCRR-	16	>128	0.1 ± 0.2
SAP-6	RCRCVCRRRKCLCQCR--	4	64	0.2 ± 0.1
SAP-7	-CRCVCRRRKCLCQC---	4	128	0.2 ± 0.1
SAP-8	RCRCVCRRRKCLCQCRR-^{A}	8	32	1.7 ± 0.1
SAP-9	-CRCVCRRRKCLCQCRRT^{A}	32	>128	-0.1 ± 0.2
SAP-10	-CRCVCRRRKCLCQCRR-^{A}	4	64	0 ± 0.1
SAP-11	RCRCVCRRRKCLCQCR--^{A}	2	16	0.5 ± 0.0
SAP-12	-CRCVCRRRKCLCQC---^{A}	2	64	-0.1 ± 0.1
SAP-13	RCRCVCRRRKCLCQCRRT	8	128	0.2 ± 0.0
SAP-14	RCRCVCRRRKCLCQCRRT	4	128	29.1 ± 0.5
SAP-15	RCRCVCRRRKCLCQCRRT	>32	>128	-0.1 ± 0.0
SAP-16	dCRCVCRRRKCLCQCddT	2	64	0 ± 0.1
SAP-17	RCRCVCddddCLCQCRRT	2	32	-0.1 ± 0.0
SAP-18	dCdCVCddddCLCQCddT	4	32	0 ± 0.0
SAP-19	oCRCVCRRRKCLCQCooT	2	64	-0.1 ± 0.1
SAP-20	RCRCVCooooCLCQCRRT	2	32	0 ± 0.1
SAP-21	oCoCVCooooCLCQCooT	8	128	0 ± 0.0
SAP-22	pCRCVCRRRKCLCQCppT	4	64	0 ± 0.1
SAP-23	RCRCVCppppCLCQCRRT	8	>128	0 ± 0.0
SAP-24	pCpCVCppppCLCQCppT	4	128	-0.2 ± 0.1
SAP-25	^{OcA}RCRCVCRRRKCLCQCR--^{A}	16	64	7.2 ± 0.4
SAP-26	RCRCVCddddCLCQCR--^{A}	2	16	-0.2 ± 0.0
SAP-27	RCRCVCRRRKCLCQCR--	>32	>128	nd

MIC: minimum inhibitory concentration, MBC: minimum bactercidal information, MH: Meuller-Hinton, HS: human serum, %Hemo: percent hemolysis at 128 µg/ml, error is one standard deviation of triplicate samples, ^{A}: amidation, Underline: D-enantiomer, d: diaminobutyric acid, o: ornithine, p: diamino proprionic acid, ^{OcA}: octanoic acid

Table S5. SAP-26 spectrum of antibacterial activity

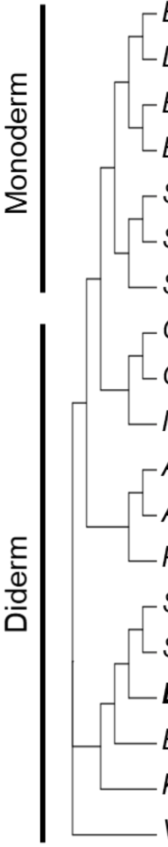

Bacterial strain	SAP-26 MH MIC (µg/ml)
Enterococcus faecium AR01	32
Listeria monocytogenes ATCC BAA-679	2
Bacillus cereus ATCC 14579	32
Bacillus subtilis PY79	1
Staphylococcus aureus USA100	32
Staphylococcus aureus ATCC 43300	32
Staphylococcus epidermidus ATCC 12228	4
Corynebacterium psuedodiphtheriticum ATCC 10700	4
Corynebacterium striatum ATCC 6940	0.5
Mycobacterium smegmatis ATCC 700084	32
Acinetobacter baumannii AB5075	64
Acinetobacter baumannii ATCC AYE	16
Pseudomonas aeruginosa ATCC 27853	8
Salmonella typhimurium LT2	4
Shigella flexneria SA100	4
***Escherichia coli* ATCC 25922**	2
Enterobacter cloacae ATCC 13047	>64
Klebsiella pneumoniae MKP103	>64
Vibrio cholerae C6706	8

MH = Mueller-Hinton; MIC = minimum inhibitory concentration

Table S6. Plasmids and Oligonucleotides.

Plasmids	Source
pMMBEH67_lpp_ompA	(16)
pDM1_empty	(32)
pDM1_mcr-1	(32)
pUltraGFP	(38)
**Oligonucleotides**	**Sequence**
oJR557 - F BH library	gtattggtaccagtcaagagcctg
oJR598 - R BH library	ctg cag gtc gac tta TBT TCV TCY MYA GWA GCA TMG GCR THT TCY CCT TMY AYA AAC GCA TYT GCV CCT ggt tcc tcc gat acc cgc ag
2x(NR)tether gBlock	ATTGCCGATGGTACACGTCAAGTCAAGAGCCTGCAGCGCCCGCCGCAG AGGCGACTCCTGCTGCTGAAGCTCCAGCTAGCGAAGCGCCTGCAGCAG AAGCTGCCCCAGCGGATGCTGCCGAAGCCCCAGCCGCTGGCATCAGTC AGGAACCTGCTGCACCAGCTGCGGAAGCTACACCAGCAGCGGAGGCAC CAGCGAGTGAAGCACCGGCTGCGGAAGCCGCTCCTGCAGATGCCGCT GAGGCTCCAGCTGCGGGTATCGGAGGAACCCGCGGTGGGCGTCTTTGT TA
F amplicon	aatgATACGGCGACCACCGAGATCTACACTCTTTCCCTACACGACGCTCT TCCGATCTCTCCAGCTGCGGGTATCGGAGGA
R index 1	CAAGCAGAAGACGGCATACGAGATCGTGATGTGACTGGAGTTCAGACG TGTGCTCTTCCGATCTgccaagcttgcatgcctgcaggtcgacTTA
R index 2	CAAGCAGAAGACGGCATACGAGATACATCGGTGACTGGAGTTCAGACG TGTGCTCTTCCGATCTgccaagcttgcatgcctgcaggtcgacTTA
R index 3	CAAGCAGAAGACGGCATACGAGATGCCTAAGTGACTGGAGTTCAGACG TGTGCTCTTCCGATCTgccaagcttgcatgcctgcaggtcgacTTA
R index 4	CAAGCAGAAGACGGCATACGAGATTGGTCAGTGACTGGAGTTCAGACG TGTGCTCTTCCGATCTgccaagcttgcatgcctgcaggtcgacTTA
R index 5	CAAGCAGAAGACGGCATACGAGATCACTGTGTGACTGGAGTTCAGACGT GTGCTCTTCCGATCTgccaagcttgcatgcctgcaggtcgacTTA
R index 6	CAAGCAGAAGACGGCATACGAGATATTGGCGTGACTGGAGTTCAGACG TGTGCTCTTCCGATCTgccaagcttgcatgcctgcaggtcgacTTA
R index 7	CAAGCAGAAGACGGCATACGAGATGATCTGGTGACTGGAGTTCAGACG TGTGCTCTTCCGATCTgccaagcttgcatgcctgcaggtcgacTTA
R index 8	CAAGCAGAAGACGGCATACGAGATTCAAGTGTGACTGGAGTTCAGACGT GTGCTCTTCCGATCTgccaagcttgcatgcctgcaggtcgacTTA
R index 9	CAAGCAGAAGACGGCATACGAGATCTGATCGTGACTGGAGTTCAGACGT GTGCTCTTCCGATCTgccaagcttgcatgcctgcaggtcgacTTA
R index 10	CAAGCAGAAGACGGCATACGAGATAAGCTAGTGACTGGAGTTCAGACGT GTGCTCTTCCGATCTgccaagcttgcatgcctgcaggtcgacTTA
R index 11	CAAGCAGAAGACGGCATACGAGATGTAGCCGTGACTGGAGTTCAGACG TGTGCTCTTCCGATCTgccaagcttgcatgcctgcaggtcgacTTA
R index 12	CAAGCAGAAGACGGCATACGAGATTACAAGGTGACTGGAGTTCAGACGT GTGCTCTTCCGATCTgccaagcttgcatgcctgcaggtcgacTTA

All oligonucleotides were ordered from Integrated DNA technologies (IDT). IDT single letter nucleotide base notations are used.

Table S7: Strains used.

Strains	Source
E. coli W3110	Lab Stock
E. coli 25922	Lab Stock
Enterococcus faecium AR01	Lab Stock
Listeria monocytogenes ATCC BAA-679	Lab Stock
Bacillus cereus ATCC 14579	Lab Stock
Bacillus subtilis PY79	Lab Stock
Staphylococcus aureus USA100	Lab Stock
Staphylococcus aureus ATCC 43300	Lab Stock
Staphylococcus epidermidus ATCC 12228	Lab Stock
Corynebacterium psuedodiphtheriticum ATCC 10700	Lab Stock
Corynebacterium striatum ATCC 6940	Lab Stock
Mycobacterium smegmatis ATCC 700084	Lab Stock
Acinetobacter baumannii AB5075	Lab Stock
Pseudomonas aeruginosa ATCC 27853	Lab Stock
Salmonella typhimurium LT2	Lab Stock
Shigella flexneria SA100	Lab Stock
Escherichia coli ATCC 25922	Lab Stock
Enterobacter cloacae ATCC 13047	Lab Stock
Klebsiella pneumoniae MKP103	Lab Stock
Vibrio cholerae C6706	Lab Stock
